# Argyrophilic nucleolar organizer region in MIB-1 positive cells in non-small cell lung cancer: clinicopathological significance and survival

**DOI:** 10.7497/j.issn.2095-3941.2014.04.005

**Published:** 2014-12

**Authors:** Dmitriy Sergeevich Kobyakov, Ashot Merudzhanovich Avdalyan, Aleksandr Fedorovich Lazarev, Elena Leonidovna Lushnikova, Lev Moiseevich Nepomnyashchikh

**Affiliations:** ^1^Budget Institution, Kogalym City Hospital, Kogalym 628484, Russia; ^2^Laboratory of Molecular Diagnostics of Altai Branch of Russian N.N. Blokhin Cancer Research Center, Barnaul 656049, Russia; ^3^Federal State Budget Institution Research Institute of Regional Pathology and Pathomorphology of Siberian Branch of Russian Academy of Medical Sciences, Novosibirsk 630117, Russia

**Keywords:** Argyrophilic nucleolar organizer region (AgNOR), MIB-1, survival, non-small cell lung cancer (NSCLC)

## Abstract

**Objective:**

To evaluate the relation between argyrophilic nucleolar organizer region (AgNOR)-associated proteins and clinicopathological parameters and survival in non-small-cell lung cancer (NSCLC).

**Methods:**

A total of 207 surgical specimens diagnosed as NSCLC were included in this study. Double-staining procedures were performed using antigen Ki-67 (clone MIB-1) and silver nitrate by immunohistochemical and AgNOR-staining methods.

**Results:**

The AgNOR area in MIB-1-positive cells of NSCLC is related to clinicopathological parameters under the TNM (tumor, node, and metastasis) system. The survival of patients with small AgNOR area in MIB-1-positive cells is better than that of patients with large AgNOR area. Molecular, biological (AgNOR area in MIB-1-positive cells), and clinicopathological (greatest tumor dimension, metastases to regional lymph nodes, histology, and differentiation) parameters are independent prognostic factors of NSCLC.

**Conclusion:**

The AgNOR area in MIB-1-positive cells is related to clinicopathological parameters and survival in NSCLC.

## Introduction

Biomarkers related to significant clinicopathological parameters of non-small cell lung cancer (NSCLC), particularly survival of patients and ability to predict disease course with a high degree of probability, should be investigated^1^. Proliferation is a basic process that reveals tumor appearance and development; this process is also a factor in the prediction of the biological behavior of tumors. However, reliable evaluation of the proliferative potential of tumors is difficult to achieve because proliferation involves not only a number of proliferative cells (proliferative activity and growth fraction) but also the velocity of cells undergoing phases of cell cycle (duration of cell cycle)^2^.

Immunohistochemical assay with Ki-67 antigen is among the most common and available methods to estimate tumor proliferative activity. Ki-67 antigen is detected in cells in late G_1_, S, G_2_, and М phases; however, the functional role of this nuclear protein in proliferation remains unclear^3^. Argyrophilic proteins associated with nucleolar organizer regions (AgNOR) are markers of cell cycle velocity. Nucleolar organizer regions are ribosomal DNA sequences on the short arms of human acrocentric chromosomes (13, 14, 15, 21, and 22) and encode ribosomal RNA (rRNA). A peculiar group of acidic and highly argyrophilic proteins are also localized at the same sites as nucleolar organizer regions, thereby allowing these regions to be very clearly and rapidly visualized by silver nitrate staining procedures. Up to 75% staining of AgNOR consists of two major argyrophilic proteins, namely, С23 (nucleolin) and В23 (nucleophosmin). Nucleolin is a 105 kDa phosphoprotein that plays an important role in the transcription of rRNA molecules; nucleophosmin is a 38 to 39 kDa phosphoprotein involved in late stages of pre-ribosomal particle organization^2^. Nucleolin and nucleophosmin are detected in cell nuclei during the entire cell cycle; these phosphoproteins increase in quantity by 1.5- to 3-fold in S and G_2_ phases^4^. Notably, the amount of AgNOR in the interphase is related to the speed of cell proliferation. AgNOR is inversely related to cell cycle duration and tumor-doubling time^5^. The relationship between interphase AgNOR quantity and cell doubling time can be attributed to proliferating cells that produce an adequate ribosomal complement for daughter cells; a short cell cycle indicates high ribosomal biogenesis per time unit. The amount of AgNOR in the interphase is also related to cell-doubling time because the number of AgNOR in the interphase is related to rRNA transcriptional activity. The evaluation of the quantitative distribution of AgNOR represents a unique tool to obtain information regarding the proliferation rate of tumors during diagnosis from histological sections of routinely processed tissue samples^2^.

Munakata *et al*.^6^ proposed the method of double staining involving Ki-67 antigen and AgNOR to evaluate AgNOR (cell cycle duration) in proliferating cells. However, limited research has been conducted regarding the proliferative potential of tumors by double staining of Ki-67 antigen and AgNOR^7^^-^^13^. Studies have also been performed regarding the importance of AgNOR^14^^-^^19^. Despite these studies, the results of double staining of Ki-67 antigen and AgNOR have not yet been evaluated by computer image analysis and in relation to clinicopathological parameters under TNM system and survival in NSCLC. The TNM system is described as follows. The T status reflects tumor size and collection of size-independent tumor descriptors, such as visceral pleural invasion, main bronchus involvement, atelectasis, or obstructive pneumonitis. The N status describes regional lymph nodes. The M status shows distant metastasis. Therefore, the current study aimed to explore the AgNOR area in MIB-1-positive cells in relation to clinicopathological parameters and survival in NSCLC.

## Materials and methods

We studied 207 surgical specimens of NSCLC resected from 2007 to 2009 in the Altai Krai Oncology Dispensary. The mean age of patients was 59 years (range, 35-75 years); the patients included 177 males (86%) and 30 females (14%). Lobectomy and pneumonectomy were performed in 145 (70%) and 62 (30%) patients, respectively. Preoperative chemotherapy and radiation therapy were not conducted. Postoperative chemotherapy, most frequently with cisplatin and etoposide, was administered to 30 patients (14%). Postoperative radiation therapy, with a total focal dose ranging from 50 to 60 Gr, was conducted in 64 patients (31%). The clinicopathological parameters of NSCLC were determined in accordance with the TNM classification of seven reviews^20^. In the current study, no cases were found with M_1_ status under the TNM system, but cases with multiple tumors were detected. The greatest tumor dimension was measured (in cm). This study was examined and approved by the corresponding ethics committee; this study was also performed in accordance with the ethical standards presented in the Declaration of Helsinki. Written informed consent was obtained.

Tissue fragments were fixed for 18 to 24 h in 10% neutral buffered formalin. After standard processing of the surgical material, we prepared histological slices of 4 µm in thickness. Specimens were stained with hematoxylin and eosin to confirm the original pathological diagnosis. For differential diagnostic purposes, histochemical (periodic acid-Schiff-alcian blue and according to Kreiberg) and immunohistochemical staining were applied. Immunohistochemical staining was performed using Ventana Discovery XT automated stainer, as described by the manufacturer (Ventana Medical System, Tucson, AZ, USA). Primary antibodies were used for cytokeratins 7 (clone SP52), 20 (clone SP33), high molecular weight (clone 34βE12), and 5/6 (clone D5/16B4). The epidermis and the gastric mucosa were used as stain control.

Based on the review of corresponding histological specimens, three tissue cores were obtained from each patient by using paraffin blocks with a needle of 1.5 mm in internal diameter. Three tissue microarrays were prepared, each containing 12×18 cores. Histological slices of 4 µm in thickness were obtained from the tissue microarrays. Slices from the tissue microarrays were immunohistochemically stained following the manufacturer’s protocol for DAKO: streptavidin–biotin method with primary antibodies to Ki-67 (clone MIB-1, DAKO) and chromogen as new fuchsin. The slices were autoclaved at 120 °C for 20 min in 0.01 М citrate buffer (рН=6.0) before staining was performed. The slices were subsequently incubated with chromogen and washed in bidistilled water. The slices were then stained with silver nitrate by one-step method^6^ in a humid chamber at 37 °C for 19 min. Further staining of nuclei was not performed, and the slices were placed in a water medium (Faramount, DAKO). Only one representative core from each patient was analyzed. Silver-stained specimens were examined under OLYMPUS CX-41 microscope equipped with a Plan C ×100/1.25 oil lens. The images were digitized and transferred directly into the computer with OLYMPUS DP72 digital camera and cellSense v.1.1 software with resolution of 1,360×1,024 pixels. The AgNOR area (in µm^2^) in each nucleus of 100 random MIB-1-positive cells (from 10 to 30 digital images) was measured. Semi-automated image processing was performed with ImageJ v.1.42. The images were subjected to conversion into 8-bit image, background, normalization, segmentation, binary processing, and final measurements. To eliminate measurement errors, we excluded granules with a size of <0.1 µm^2^ from analysis. The internal control of stromal lymphoid follicles was attended by MIB-1-positive centroblasts (positive control) and MIB-1-negative small lymphocytes (negative control). The entire AgNOR quantification was performed by one of the authors.

### Statistical analysis

Statistical analysis was performed using STATISTICA 6.0. Data values were expressed as median and interquartile range. Data were processed by Kruskal-Wallis test, and significant differences were assessed by Mann–Whitney U test. Comparisons were performed using χ^2^ test. Probabilities of 5-year overall survival were calculated by Kaplan-Meier method (in %), and between-group comparisons were conducted using log-rank test. Univariate and multivariate Cox proportional hazards model was used for the following factors: age at surgery (<59 *vs*. ≥59 years), gender (male *vs*. female), type of surgery (lobectomy *vs*. pneumonectomy), postoperative chemotherapy (yes *vs*. no), postoperative radiation therapy (yes *vs*. no), T status (T_1_ *vs*. T_2_ to T_3_), greatest tumor dimension (<3 *vs*. ≥3 cm), N status (N_0_ *vs*. N_1_ to N_3_), TNM stage (I *vs*. II to III), histology (adenocarcinoma *vs*. squamous-cell cancer), and differentiation (well *vs*. moderate to poor). Statistical significance was set at *P*<0.05.

## Results

**Figure 1:**
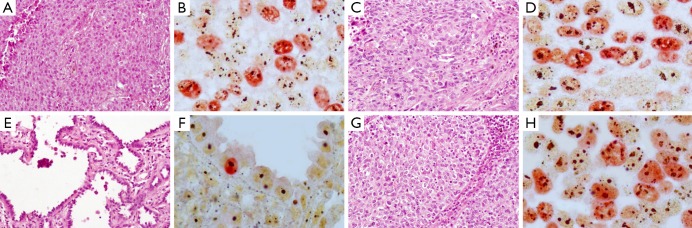
AgNOR in MIB-1-positive and MIB-1-negative cells of NSCLC in squamous cell cancer with absence (А, B) and presence (C, D) of metastases to lymph nodes and in adenocarcinoma with well (E, F) and poor (G, H) differentiation. Double staining for Ki-67 (clone MIB-1) by immunohistochemistry and for AgNOR with silver nitrate, ×1,000. AgNOR, argyrophilic nucleolar organizer region; NSCLC, non-small-cell lung cancer.

Immunohistochemical staining results of slides with primary antibodies for MIB-1 and subsequent staining results with silver nitrate were detected in the form of black round granules (AgNOR) located against a red nucleus (MIB-1-positive cells) or against a brown nucleolus or pale yellow nucleus (MIB-1-negative cells; **Figure 1**). In the nuclei, isolated black round granules (dots) appeared in the nucleoplasm (usually in MIB-1-negative cells) and/or multiple black round granules (dots) as a cluster (usually in MIB-1-positive cells). The morphometric results of the AgNOR area in MIB-1-positive cells in different clinicopathological parameters of NSCLC are shown in **Table 1**.

**Table 1:** AgNOR area in MIB-1-positive cells and clinicopathological parameters of NSCLC

Clinicopathological parameters	*n* (%)	AgNOR area (in µm^2^)	*P*
T status			<0.001
T_1_	55 (27)	8.95 (7.60 to 10.91)	
T_2_ to T_3_	152 (73)	11.08 (8.92 to 13.16)	
Greatest tumor dimension			<0.001
<3 cm	87 (42)	8.93 (7.60 to 10.92)	
≥3 cm	120 (58)	11.60 (9.37 to 13.42)	
N status			<0.001
N_0_	132 (64)	9.43 (8.04 to 11.88)	
N_1_ to N_3_	75 (36)	11.89 (10.37 to 14.23)	
TNM stage			<0.001
I	107 (52)	9.35 (7.89 to 11.87)	
II to III	100 (48)	11.41 (9.21 to 13.75)	
Histology			0.8
Adenocarcinoma	94 (45)	10.20 (8.79 to 12.53)	
Squamous cell cancer	113 (55)	10.58 (8.37 to 12.69)	
Differentiation			<0.001
Well	52 (25)	8.79 (7.73 to 10.95)	
Moderate to poor	155 (75)	11.01 (8.97 to 13.04)	

*n*, number of cases; %, percentage of cases; T, T status under TNM system; N, N status under TNM system; *P*, *P* value (statistical significance). AgNOR, argyrophilic nucleolar organizer region; NSCLC, non-small-cell lung cancer.

In NSCLC, the median AgNOR area in MIB-1-positive cells was 10.47 µm^2^ (range, 8.57-12.69 µm^2^), and this value was chosen as cut-off point. Cases with AgNOR area ≥10.47 µm^2^ in MIB-1-positive cells were counted as large-area cases (101 cases, 49%) and <10.47 µm^2^ as small-area cases (106 cases, 51%). The AgNOR area in MIB-1-positive cells was significantly larger in groups T_2_ and T_3_ than in T_1_. In NSCLC with the greatest tumor dimension of <3 cm, the AgNOR area in MIB-1-positive cells was smaller than in those with tumor size ≥3 cm. A statistically significant increase in the AgNOR area in MIB-1-positive cells of NSCLC with metastases to regional lymph nodes was observed versus non-metastatic tumors (**Figure 1A-D**). The AgNOR area in MIB-1-positive cells was smaller in TNM stage I than in stages II to III. No difference was observed in the AgNOR area in MIB-1-positive cells of adenocarcinoma and squamous cell cancer. The AgNOR area in MIB-1-positive cells was larger in moderately and poorly differentiated tumors than in well-differentiated tumors (**Figure 1E-H**). The AgNOR area in MIB-1-positive cells of NSCLC was correlated with Т status (*P*<0.001), greatest tumor dimension (*P*<0.001), N status (*P*<0.001), TNM stage (*P*<0.001), and differentiation (*P*<0.001).

**Figure 2:**
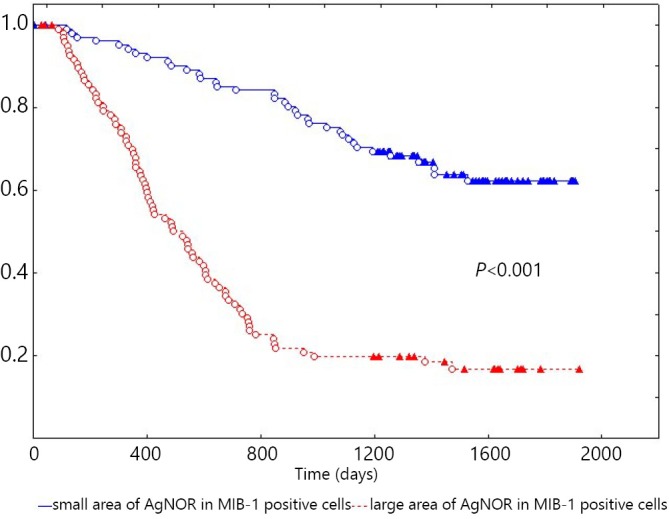
Kaplan-Meier curve of patients with NSCLC with small and large AgNOR areas in MIB-1-positive cells. X-axis shows the time of life (in days); Y-axis shows the proportion of surviving patients. NSCLC, non-small-cell lung cancer; AgNOR, argyrophilic nucleolar organizer region.

The 5-year overall survival of patients with NSCLC was (39.3±3.8)%. The overall survival of patients with NSCLC exhibited statistically significant difference (*P*<0.001) based on the AgNOR area in MIB-1-positive cells at (61.2±5.4)% in small area *vs*. (16.2±4.2)% in large area (**Figure 2**). The overall survival of patients with NSCLC was also stratified based on the AgNOR area in MIB-1-positive cells, and the clinicopathological parameters showed statistically significant difference (**Table 2**). In univariate analysis, the following factors affected the survival of patients: AgNOR area in MIB-1-positive cells (χ^2^=59.9, *P*<0.001), N status (χ^2^=52.2, *P*<0.001), TNM stage (χ^2^=44.2, *P*<0.001), differentiation (χ^2^=21.7, *P*<0.001), greatest tumor dimension (χ^2^=21.3, *P*<0.001), type of surgery (χ^2^=8.7, *P*=0.002), T status (χ^2^=6.9, *P*=0.01), and histological characteristics (χ^2^=5.2, *P*=0.02). Age, gender, postoperative chemotherapy, and radiation therapy did not affect the survival of patients with NSCLC in univariate analysis. In multivariate analysis (χ^2^=120.2), the survival of patients was affected by the AgNOR area in MIB-1-positive cells (β=1.05, standard error =0.23, *P*<0.001), greatest tumor dimension (β=0.94, standard error =0.35, *P*=0.007), metastases to regional lymph nodes (β=0.79, standard error =0.34, *P*=0.02), histology (β=0.23, standard error =0.11, *P*=0.03), and differentiation (β =0.66, standard error =0.29, *P*=0.02). Likewise, age, gender, type of surgery, postoperative chemotherapy, radiation therapy, T status, and TNM stage did not influence the survival of patients with NSCLC in multivariate analysis.

**Table 2:** Five-year overall survival stratified according to cut-off point of AgNOR area in MIB-1-positive cells and clinicopathological parameters of NSCLC

Clinicopathological parameters	AgNOR area, *n* (%)		*Р*
	<10.47 µm^2^	≥10.47 µm^2^	
T status			
T_1_	40 (54.9±9.3)	15 (22.9±12.1)	0.006
T_2_ to T_3_	66 (63.1±6.7)	86 (14.7±4.2)	<0.001
Greatest tumor dimension			
<3 cm	62 (63.9±7.2)	25 (26.5±9.2)	<0.001
≥3 cm	44 (54.3±8.8)	76 (12.5±4.3)	<0.001
N status			
N_0_	87 (63.6±5.9)	45 (34.7±8.5)	<0.001
N_1_ to N_3_	19 (37.1±15.6)	56 (0)	<0.001
TNM stage			
I	73 (65.4±6.4)	34 (37.6±10.0)	0.002
II to III	33 (46.8±11.0)	67 (4.2±2.7)	<0.001
Histology			
Adenocarcinoma	50 (45.0±9.0)	44 (7.9±4.8)	<0.001
Squamous cell cancer	56 (72.3±6.7)	57 (19.6±6. 2)	<0.001
Differentiation			
Well	37 (68.6±9.2)	15 (44.4±13.5)	0.03
Moderate to poor	69 (54.9±6.8)	86 (10.9±3.7)	<0.001

*n*, number of cases; T, T status under TNM system; N, N status under TNM system; *P*, *P* value (statistical significance). AgNOR, argyrophilic nucleolar organizer region; NSCLC, non-small-cell lung cancer.

## Discussion

Our study found the relationship between the AgNOR area in MIB-1-positive cells of NSCLC and the following clinicopathological parameters under the TNM system: T status, greatest tumor dimension, N status, TNM stage, and differentiation. Other studies have also shown the relation of individual clinicopathological parameters under the TNM system with AgNOR in MIB-1-positive cells. Yamaguchi^13^ found that AgNOR increases in MIB-1-positive cells of NSCLC in Т_4_ *vs*. Т_1_ to T_3_ and in N_2_ to N_3_ *vs*. N_0_ to N_1_. Kidogawa *et al*.^10^ also found that AgNOR increases in MIB-1-positive cells of breast cancer with the largest size of >2 cm *vs*. tumor size <2 cm. Tomobe *et al*.^12^ found a sequential increase in AgNOR in MIB-1-positive cells of bladder cancer in Т_1_, Т_2_, and Т_3_. In contrast to our current results obtained from objective analysis (computer image analysis and measurement of AgNOR area), these previous results were obtained from subjective analysis (visual counting of AgNOR number). Other studies have shown the relationship between clinicopathological parameters under TNM system and AgNOR^14^^-^^18^. Thus, our study showed that the AgNOR area in MIB-1-positive cells was correlated with clinicopathological parameters, thereby reflecting the relationship among molecular, biological, and clinicopathological parameters of NSCLC.

The survival of patients with NSCLC with small AgNOR area in MIB-1-positive cells is better than that of those with greater AgNOR area, as well as in homogeneous groups of clinicopathological parameters. In univariate and multivariate regression analyses, the AgNOR area in MIB-1-positive cells independently affected the survival of patients with NSCLC. Our results correlate with those of studies on NSCLC^9^, breast cancer^7^^,^^8^^,^^10^^,^^11^, and bladder cancer^12^. Studies on AgNOR in malignant tumors have also revealed that the content of AgNOR is an independent prognostic factor^16^^-^^19^. The prognostic value of AgNOR in MIB-1-positive cells is related to different rates of NSCLC proliferation. A large AgNOR area in MIB-1-positive cells indicates short cell cycle of proliferating cells and high proliferation speed. By contrast, small area implies long cell cycle of proliferating cells and low proliferation speed. Thus, our study showed that the AgNOR area in MIB-1-positive cells was correlated with the survival of patients with NSCLC; this result revealed the relationship of molecular and biological parameters, as well as biological behaviors of tumors. The current results provide further insights into an accurate assessment of actual survival curves and prognosis of patients with NSCLC.

## Conclusion

In NSCLC, clinicopathological parameters under the TNM system are related to molecular and biological parameters, including AgNOR area in MIB-1-positive cells. The survival of patients with NSCLC with a small AgNOR area in MIB-1-positive cells is better than that of patients with a large AgNOR area. Molecular, biological (AgNOR area in MIB-1-positive cells), and clinicopathological (greatest tumor dimension, metastases to regional lymph nodes, histology, and differentiation) parameters are independent prognostic factors of NSCLC.

## References

[1] Larsen JE Minna JD. Molecular biology of lung cancer: clinical implications. Clin Chest Med 2011;32:703-740. 22054881 10.1016/j.ccm.2011.08.003 PMC3367865

[2] Derenzini M. The AgNORs. Micron 2000;31:117-120. 10588056 10.1016/s0968-4328(99)00067-0

[3] Bullwinkel J Baron-Lühr B Lüdemann A Wohlenberg C Gerdes J Scholzen T. Ki-67 protein is associated with ribosomal RNA transcription in quiescent and proliferating cells. J Cell Physiol 2006;206:624-635. 16206250 10.1002/jcp.20494

[4] Sirri V Roussel P Hernandez-Verdun D. The AgNOR proteins: qualitative and quantitative changes during the cell cycle. Micron 2000;31:121-126. 10588057 10.1016/s0968-4328(99)00068-2

[5] Canet V Montmasson MP Usson Y Giroud F Brugal G. Correlation between silver-stained nucleolar organizer region area and cell cycle time. Cytometry 2001;43:110-116. 11169575 10.1002/1097-0320(20010201)43:2<110::aid-cyto1025>3.0.co;2-7

[6] Munakata S Hendricks JB. A multilabeling technique for simultaneous demonstration and quantitation of Ki-67 and nucleolar organizer regions (AgNORs) in paraffin-embedded tissue. J Histochem Cytochem 1994;42:789-793. 7514625 10.1177/42.6.7514625

[7] Abboud P, Lorenzato M, Joly D, Quereux C, Birembaut P, Ploton D. Prognostic value of a proliferation index including MIB1 and argyrophilic nucleolar organizer regions proteins in node-negative breast cancer. Am J Obstet Gynecol 2008;199:146,e1-146,e7. 10.1016/j.ajog.2008.02.025 18455135

[8] Biesterfeld S Farokhzad F Klüppel D Schneider S Hufnagl P. Improvement of breast cancer prognostication using cell kinetic-based silver-stainable nucleolar organizer region quantification of the MIB-1 positive tumor cell compartment. Virchows Arch 2001;438:478-484. 11407476 10.1007/s004280000351

[9] Bigras G Marcelpoil R Brambilla E Brugal G. Interest of targeting AgNORs measurement in cycling cells: in vivo cell kinetic evaluation of non-small cell lung cancer. Anal Cell Pathol 1996;11:183-198. 8888954

[10] Kidogawa H Nanashima A Yano H Matsumoto M Yasutake T Nagayasu T. Clinical significance of double staining of MIB-1 and AgNORs in primary breast carcinoma. Anticancer Res 2005;25:3957-3962. 16309183

[11] Lorenzato M Abboud P Lechki C Browarnyj F O’Donohue MF Ploton D Proliferation assessment in breast cancer: a double-staining technique for AgNOR quantification in MIB-1 positive cells especially adapted for image cytometry. Micron 2000;31:151-159. 10588061 10.1016/s0968-4328(99)00072-4

[12] Tomobe M Shimazui T Uchida K Hinotsu S Akaza H. Argyrophilic nucleolar organizer region in proliferating cell has a predictive value for local recurrence in superficial bladder tumor. J Urol 1999;162:63-68. 10379741 10.1097/00005392-199907000-00016

[13] Yamaguchi S. Relationship between the responses to simultaneous double staining for Ki-67 and AgNOR and the clinicopathological features of non-small cell pulmonary carcinoma. Acta Med Nagasaki 1994;39:147-152.

[14] Eröz R Unluhizarci K Cucer N Ozturk F. Value of argyrophilic nucleolar organizing region protein determinations in nondiagnostic fine needle aspiration samples (due to insufficient cell groups) of thyroid nodules. Anal Quant Cytopathol Histpathol 2013;35:226-231. 24341126

[15] Eroz R Cucer N Unluhizarci K Ozturk F. Detection and comparison of cut-off values for total AgNOR area/nuclear area and AgNOR number/nucleus in benign thyroid nodules and normal thyroid tissue. Cell Biol Int 2013;37:257-261. 23364912 10.1002/cbin.10038

[16] Winzer KJ Bellach J Hufnagl P. Long-term analysis to objectify the tumour grading by means of automated microscopic image analysis of the nucleolar organizer regions (AgNORs) in the case of breast carcinoma. Diagn Pathol 2013;8:56. 23566354 10.1186/1746-1596-8-56 PMC3640910

[17] Avdalyan A, Bobrov I, Klimachev V, Lazarev A. Prognostic Value of Microvessel Density in Tumor and Peritumoral Area as Evaluated by CD31 Protein Expression and Argyrophilic Nucleolar Organizer Region Count in Endothelial Cells in Uterine Leiomyosarcoma. Sarcoma 2012;2012:594512. 10.1155/2012/594512 PMC3403075 22910809

[18] Treré D Ceccarelli C Migaldi M Santini D Taffurelli M Tosti E Cell proliferation in breast cancer is a major determinant of clinical outcome in node-positive but not in node-negative patients. Appl Immunohistochem Mol Morphol 2006;14:314-323. 16932023 10.1097/00129039-200609000-00010

[19] Pich A Chiusa L Margaria E. Prognostic relevance of AgNORs in tumor pathology. Micron 2000;31:133-141. 10588059 10.1016/s0968-4328(99)00070-0

[20] Sobin LH, Gospodarowicz MK, Wittekind C. eds. TNM classification of malignant tumours. 7th edition. Oxford: Wiley-Blackwell, 2009.

